# Transport through molecular junctions

**DOI:** 10.3762/bjnano.2.74

**Published:** 2011-10-18

**Authors:** Jan M van Ruitenbeek

**Affiliations:** 1 Kamerlingh Onnes Laboratory, Leiden Institute of Physics, Leiden University, Niels Bohrweg 2, 2333 CA Leiden, The Netherlands

Exploiting the rich design space of organic molecules for applications in future electronic devices is one of the main challenges in nanotechnology. Several groups have recently demonstrated, for a limited set of molecules, clear single-molecule characteristics and fair agreement with computations. Now that attaching leads to individual molecules has been demonstrated we naturally enter into the next exciting phase of the research, where molecule-specific properties can be engineered and studied.

The most prominent property that distinguishes organic molecules from inorganic quantum dots and nanowires is that they are floppy nano-objects with a strong coupling between charge transport and vibrational degrees of freedom (vibrons). This coupling is predicted to influence transport in dramatic ways as it may destroy the coherence of charge carriers on the molecules and can lead to strong nonequilibrium effects. Many exciting predictions have been discussed in the recent literature showing unique features of single-molecule junctions. These properties can be designed and controlled by chemists.

Studying them experimentally requires creating an interface between the molecules and at least two metallic leads. Standard nanofabrication techniques fall short by more than an order of magnitude in the distance and precision required for addressing molecules, which typically have a length of the order of one nanometre. Several methods have now been established that can meet the requirements, of which three are particularly prominent: The electrode separation can be mechanically adjusted at will, through piezoelectric actuators, in a setup based on a scanning tunnelling microscope. Alternatively, this can be achieved by mechanically controllable break junctions. A third, widely used method employs breaking of a thin wire by electromigration. Many methods have been explored for introducing the molecules into the junction, but in all cases there is an element of chance, and variations in the attachment of the molecule in the nanogap between the electrodes are common. It is now widely recognized that the variability in bonding, which is most clearly observed through the variability in conductance, is part of the physics of the problem and inherent to (single) molecular junctions. The study of such junctions requires the gathering of statistics covering many configurations.

The field of study of electron transport through molecular junctions requires input from many subdisciplines and this interdisciplinary character leads to many new initiatives and research consortia. Theoretical research is strongly represented, and is leading the experiments. This illustrates the fact that many aspects are still unexplored, but also that experiments are difficult. The past has seen some premature claims and overly enthusiastic presentations of results that have later been proven hard to reproduce. Molecules are simply very small objects, and while we are capable of visualizing molecules by various techniques, such approaches are still incompatible with molecules being directly coupled to metallic leads. Consequently, the measured results are often interpreted based upon reasonable assumptions regarding the geometry of the molecular bonding. Our imagination has often been shown to be too limited to capture all aspects of the actual molecular device. More recently, new techniques have been developed that shed further light onto the problem and help build confidence in our interpretations. Apart from direct conductance measurements, one now measures properties such as thermopower, shot noise, Raman scattering, photo-induced switching, and gate-induced level shifts, all at the single-molecule level. Moreover, by systematic variation of the chemical structure of the molecule, it is possible to map out the dominant current-carrying pathways in the molecule and to systematically explore the effects of bond angles and side groups.

Our field of study also poses new challenges to theory and computational methods. Density functional theory is well established and the limits of its validity are well known when applied to bulk metallic systems, as well as to organic molecules in isolation. However, these two quite separate application areas need to be married together, and there is the additional problem of the nonequilibrium electron distribution resulting from the applied bias. The new challenges that this brings have attracted the attention of many of the best groups in world and important progress has been made in the theoretical and computational methods.

More recently, a new element has been added to the discussion. The electron flow is known to exert a force on the ions, which is responsible for the phenomenon of electromigration mentioned above. However, the microscopic theory for the effect is poorly understood. It has recently been shown, through seminal work by Todorov and his group, that the electron force is nonconservative. This implies that the current is capable of doing work and that it should be possible to devise molecular motors that are directly driven by an electron current.

I am pleased to see that we have succeeded in bringing together, in this Thematic Series, contributions of experimental and theoretical research in physics and chemistry from some of the most prominent groups in this field. The works published in this thematic series of the Beilstein Journal of Nanotechnology illustrate the vitality of the research and the many lines along which the research is developing. Together, the papers give a snapshot of where the field stands at this moment.

Jan M. van Ruitenbeek

Leiden, October 2011

